# The Value of Ultrasonography Using Thyroid Imaging Reporting and Data Systems (TIRADS) in the Diagnosis of Thyroid Cancer Among the Population of Ha'il, Saudi Arabia

**DOI:** 10.7759/cureus.27437

**Published:** 2022-07-29

**Authors:** Ashraf Elaggan, Amr Mostafa, Raghad Albdair, Rema Almarshedi, Anas Aljohani, Zaid Alshammari

**Affiliations:** 1 Department of Diagnostic Radiology, King Salman Specialist Hospital, Ha'il, SAU; 2 Department of Diagnostic Radiology, Tanta University, Tanta, EGY; 3 Department of Diagnostic Radiology, University of Hail, Ha'il, SAU; 4 Department of Diagnostic Radiology, Taibah University, Medina, SAU; 5 Department of Diagnostic Radiology, King Fahad Medical City, Riyadh, SAU

**Keywords:** tirads, thyroid nodule, ultrasonography, cancer, thyroid, fine needle aspiration

## Abstract

Background: Thyroid cancer is one of the common malignant conditions of the head and neck region, and it is considered as one of the most common cancers among endocrine tumors. Ultrasonography is widely used in order to assess thyroid nodules, Therefore, the aim of our study is to determine the accuracy of ultrasonography and fine needle aspiration biopsy (FNAB)-guided by ultrasonography in the diagnosis of thyroid malignancy among the population in the Ha'il region of Saudi Arabia.

Methods: A retrospective evaluation of 137 patients was undertaken at King Salman Specialist Hospital, Ha'il, Kingdom of Saudi Arabia. Patients who presented with thyroid swellings underwent ultrasonography and FNAB.

Result: Our study results have concluded that the sensitivity of the ultrasonography in the confirmation of a malignant thyroid lesion is 59.4% and its specificity was found to be 74.3%.

Conclusion: The study showed that ultrasonography using Thyroid Imaging Reporting and Data Systems (TIRADS) is a significant step in evaluating a thyroid nodule. Also, it is highly sensitive and specific, cost-effective, and convenient for the patient.

## Introduction

Thyroid cancer is the most common form of endocrine cancer worldwide, with an incidence of 56 cases per 100,000 [1]. Thyroid cancer is the fifth most common cancer in females and the second most common in women older than 50 years [2]. The incidence of thyroid cancer worldwide has increased at a higher rate than any other cancer in the past several decades, while mortality rates for thyroid cancer have remained almost stable. One of the most important risk factors is exposure to ionizing in some environments, along with female gender, age, and genetics [3,4]. The clinical manifestations of thyroid cancer include thyroid nodule, difficulty in swallowing, difficulty with breathing, and hoarseness of voice [5].

For investigation, ultrasound imaging and fine-needle aspiration cytology is needed. The features of ultrasonography that are suspicious of thyroid cancer include the presence of marked hypo-echogenicity, micro-lobulated or irregular margins, micro-calcifications, and a taller-than-wide shape [6].

Thyroid cancer is diagnosed histologically through fine needle aspiration biopsy (FNAB) and is categorized into four types: (i) papillary thyroid carcinoma, which is the most common type of thyroid cancer and is the least aggressive type of cancer, because it grows and metastasis slowly; (ii) follicular thyroid carcinoma, which is almost 14% of thyroid malignancies, is more aggressive than papillary thyroid carcinoma; (iii) medullary thyroid carcinoma, a cancer of non-thyroid cells that are normally present in the thyroid gland, medullary thyroid cancer is almost 3% of thyroid malignancies; (iv) anaplastic thyroid cancer, which is almost 2% of thyroid cancers and is the most dangerous type of thyroid malignancies because it metastasizes early to the surrounding lymph nodes and also distant organs [5].

FNAB is currently the gold standard for evaluating and assessment of thyroid nodules that could be cancerous [3]. 

## Materials and methods

This study is a retrospective evaluation of a prospectively maintained data set of patients at the King Salman Specialist Hospital, Ha'il, Saudi Arabia. Data were collected from the patient’s medical records and reviewed. This data collection process was focused on patients who were admitted to the hospital with thyroid-related diseases or outpatients who visited the hospital with similar complaints. In this study, the data collected was evaluated to determine the accuracy of ultrasonography compared to FNAB guided by ultrasonography in the diagnosis of thyroid malignancy among the population in the Ha'il region in the two-year period of 2019-2020.

The authors retrospectively evaluated the electronic medical records of 137 patient subjects during the study period of October to November, 2021, in King Salman Specialist Hospital. Of the subjects, 123 were females and 14 were males. Furthermore, an assessment of the health comorbidities was done to better evaluate the subjects. The last section of the study was designed to study and analyze the outcomes of the ultrasonographical method for evaluating a thyroid lesion along with the reports of the histopathology to later find out the sensitivity and the specificity of the ultrasound in confirming thyroid malignancy. A chi-square non-parametric test was implemented to find the statistical significance of the study categorical variables, and a p-value of <0.001 was accepted to be significant. 

Imaging evaluation

An ultrasound session was conducted on each patient suspected to have a thyroid lesion after a complete clinical examination. Each of the subjects had an evaluation of whether the suspected thyroid lesion is considered benign or malignant, and a TIRADS score was given to each patient after reporting the findings.

Histopathological evaluation

Further, fine needle aspiration of the thyroid was also done for histopathological evaluation of the thyroid lesion after the ultrasonography for each subject to ultimately confirm the diagnosis of either a benign condition or a malignancy. 

## Results

Sociodemographic data

A total of 137 patient subjects’ electronic medical reports were reviewed and evaluated. Of the total, the majority were females (n= 123, 89.8%) and only 14 (10.2%) were males. The different comorbidities associated with the patients' presentation were observed and it was found that 43.8% were medically free, 27.0% had diabetes, 16.8% had hypertension, and 15.3% had hypothyroidism. Other medical conditions that were found in lesser numbers included asthma, Hodgkin’s lymphoma, mammary duct ectasia, and previous medical history of atrial fibrillation. Table 1 describes the baseline sociodemographic information and the associated comorbidities. 

**Table 1:** Baseline sociodemographic information of the subjects

Gender	N (%)
Female	123 (89.8%)
Male	14 (10.2%)
Associated Comorbidities	
Diabetes	38 (27.0%)
Hypertension	23 (16.8%)
Dyslipidemia	5 (3.6%)
Hypothyroidism	21 (15.3%)
Hyperthyroidism	4 (2.9%)
Medically free	60 (43.8%)

Ultrasonography, histopathological findings, and TIRAD scores

The authors considered any potential or confirmed thyroid malignancy on the ultrasound reports as “positive” and all the confirmed benign thyroid conditions as “negative”. The majority (n=91, 66.4%) were positive (malignant) based on the ultrasound findings, and 46 (33.6%) were negative (benign). 

Of the total, most were diagnosed as benign follicular nodules with cystic degeneration based on the histopathological studies (n= 29, 21.2%). Twenty-five (18.2%) were diagnosed as papillary thyroid carcinoma, 21 (15.3%) were benign follicular nodules, 16 (11.7%) were confirmed as adenomatoid nodules with cystic degeneration, and 10 (7.3%) were chronic lymphocytic thyroiditis. However, it is necessary to acknowledge that 22 (16.1%) were negative for evidence of malignancy (Table 2). With the use of Chi-square non-parametric test, it was found that the difference between the histopathological findings were statistically significant (p <0.001).

**Table 2:** The histopathological findings for the thyroid assessment

Histopathology Findings	N (%)
Adenomatoid nodule	1 (0.7%)
Adenomatoid nodule with cystic degeneration	16 (11.7%)
Adenomatoid nodule with cystic degeneration (suspicious for follicular cancer	1 (0.7%)
Benign colloid nodule	5 (36%)
Benign follicular nodule	21 (15.3%)
Benign follicular nodule with cystic degeneration	29 (21.2%)
Chronic lymphocytic thyroiditis	10 (7.3%)
Follicular neoplasm	7 (5.1%)
Negative for malignancy	22 (16.1%)
Papillary thyroid carcinoma	25 (18.2%)

Furthermore, the authors have investigated the TIRAD scores given for each confirmed thyroid condition falling in one of the TIRAD categories (TR1, TR2, TR3, TR4, and TR5). It was noted that the majority of them were scored as TR1 (n= 46, 33.6%), while 22 (16.1%) were reported as TR2, 24 (17.5%) were reported as TR3, 29 (21.2%) were reported as TR4, and 16 (11.7%) were reported as TR5 (p <0.001).

Comparing the histopathological findings and the given TIRAD score, the authors found that the majority of those who had benign findings on histopathology scored “TR1” (n=41, 39.0%), while the majority of those who had malignant findings on histopathology scored “TR5” (n=10, 31.3%) and to a lesser extent “TR4” (n=9, 28.1%), with all findings’ association between TiRAD and results of histopathology showing statistical significance (p<0.001). Table 3 describes the different scores given for the benign and malignant findings. 

**Table 3:** The frequencies of the TIRAD scores given to each histopathological finding TIRAD: Thyroid Imaging Reporting and Data Systems

Associations between Histopathology and the TIRADS score							
		TIRADS score					
		TR1	TR2	TR3	TR4	TR5	P<0.001
Histopathology	Benign	41 (39.0%)	19 (18.1%)	19 (18.1%)	20 (19.0%)	6 (5.7%)	
	Malignant	5 (15.6%)	3 (9.4%)	5 (15.6%)	9 (28.1%)	10 (31.3%)	

Sensitivity and specificity of ultrasonography in thyroid malignancy

The sensitivity, also known as the true positive rate, of ultrasonography to confirm the diagnosis of a malignant thyroid condition was found to be 59.4%. Moreover, the false negative rate, also known as the type two error, was found to be 40.6%. In contrast, the false positive rate, also known as type one error, was found to be 25.7%. However, the specificity of the ultrasonography, also known as the true negative rate, was found to be 74.3%. 

## Discussion

Thyroid cancer is a common malignant condition of the head and neck region. The incidence of this type of malignancy is considerably high, with an incidence of approximately 56 diagnosed cases in 100,000 individuals, ranking it as the topmost common among endocrine tumors [7,8]. In the last decade, the rate of occurrence of thyroid cancer has escalated with the general environmental deterioration, increasing its threat to public health [9].

In most clinical presentations, the early signs and symptoms of thyroid malignancy are similar to the manifestations of benign thyroid nodules. The presentation of these nodules usually includes difficulty in the natural process of breathing, neck swallowing, hoarseness of the voice, and obstruction. Therefore, a clinician should focus to diagnose and possibly exclude thyroid malignancy in a patient presenting with a thyroid nodule [10,11]. The most common thyroid cancer type is papillary adenocarcinoma, while the least common type is undifferentiated carcinoma [12]. Commonly, thyroid cancer occurs unilaterally, meaning at one side of the gland, often singularly [13]. At a pathologic level, the most commonly encountered thyroid nodule is of the papillary adenocarcinoma category, accounting for approximately 70% of all the pathological types [12].

In daily clinical practice, the ultrasound imaging modality is often utilized to diagnose thyroid cancer. However, overlaps in the images recorded by the ultrasound occur as well as frequent diversity. Thereby, a high rate of misdiagnosis is notable with the use of ultrasound imaging solely in diagnosing early as well as atypical types of thyroid cancer [14]. Fortunately, FNAB is considered a safe and simple technique, with higher diagnostic accuracy and sensitivity in the process of thyroid cancer detection [15]. Nowadays, patients presenting to the clinic with thyroid nodules are often initially examined by ultrasound. After the ultrasound examination, these patients are subjected to be examined by FNAB further in the assessment. In addition, FNAB with the guidance of ultrasonography is considered to be the current gold standard method of evaluating a thyroid lesion due to the high sensitivity associated with its findings [16]. Nevertheless, the final diagnosis of some of the thyroid lesions such as follicular adenocarcinoma for instance remains unclear [17]. The accuracy of FNAB diagnosis is based on multiple variables. These variables include the technical skills of the individual performing it and the skills of the pathologist who will process and interpret the sample biopsy. 

Even though FNAB is the method of choice, the first step taken in evaluating any thyroid nodule is the use of ultrasonography. This is followed by FNAB, which decides whether the patient will undergo surgical management or not [18].

TIRADS is a criteria figured based on the radiological findings of the thyroid gland. Based on the results of the TIRADS, the decision of subjecting a lesion to FNAB is made [19]. This TIRADS algorithm used to report a thyroid lesion has clear and defined set objectives that are based on the ultrasonographic features, and the higher the TIRADS classification, the higher the thyroid malignancy risk [20,21].

In this study, the authors investigated ultrasound imaging with the use of the TIRADs criteria and FNAB method of diagnosis in relation to its diagnostic value and its role of contribution in establishing a diagnosis of thyroid malignancy. Our study results have concluded that the sensitivity of the ultrasonography in the confirmation of a malignant thyroid lesion in 59.4% and its specificity was 74.3%. In comparison, Youssef et al. have concluded in a similar study that the sensitivity of ultrasonography in detecting was 100% with a specificity of 94.12% [18].

Moreover, Wettasinghe and colleagues have noted in their published study that detecting microcalcifications as well as hypoechogenicity is considered to be the most necessary points of criteria in the prediction of thyroid malignancy, which matched our study findings [22].

Ram et al., in another study, have evaluated the sensitivity and specificity of ultrasonography in diagnosing thyroid nodules concluding that this radiological method in regard to calcifications is 80% sensitive and 68% specific, and based on hypoechogenicity are 80% and 52%, respectively [23].

Furthermore, and in comparison to our study findings, in a study conducted by Li et al. to figure the diagnostic value of FNAB in combination with the use of ultrasound, it was found that the sensitivity of the ultrasound in diagnosing thyroid cancer was 86.67%, the specificity was 28.57%, the accuracy was 80.60%, while the positive predictive value was 91.23%, and the negative predictive value was 25% [1]. Additionally, Yang et al. have noted in their study that the fine needle aspiration has an accuracy percentage of 100%; however, this accuracy is only implicated in the setting of medullary thyroid carcinoma specifically if the diameter is more than 1 cm. Moreover, if the diameter is less than 1 cm, the accuracy of the FNAB becomes 66.6% [24]. There is a published study conducted by Bahaj et al. who noted in a study on 314 subjects, the sensitivity of the fine needle aspiration is 79.8%, while the specificity was valued at 82.1%. Regarding the positive predictive value, it was found to be 74.8%, the accuracy was 84.8%, and the negative predictive value was 85.9%, which were all generally similar to our study findings [25].

Conclusively, more clinical retrospective studies should be conducted to assess the diagnostic validity of ultrasonography in detecting thyroid malignancies in order to accurately compare the different study results and asses this radiological approach.

## Conclusions

In conclusion, ultrasonography using TIRADS is a very significant step in evaluating a thyroid nodule before moving on to the next step of FNAB, despite the fact that it is the gold standard method of diagnosis. Ultrasonography is highly sensitive and specific, cost-effective, and convenient to the patient. Thereby, more retrospective studies should be conducted in the future to evaluate its diagnostic accuracy.

## References

[1] Li J, Wang Q, Wang L (2019). Diagnostic value of fine-needle aspiration combined with ultrasound for thyroid cancer. Oncol Lett.

[2] Vaccarella S, Dal Maso L (2021). Challenges in investigating risk factors for thyroid cancer. Lancet Diabetes Endo.

[3] Khodamoradi F, Ghoncheh M, Mehri A, Hassanipour S, Salehiniya H (2018). Incidence, mortality, and risk factors of thyroid cancer in the world: a review. World Cancer Research Journal.

[4] Liu Y, Su L, Xiao H (2017). Review of factors related to the thyroid cancer epidemic. Int J Endocrinol.

[5] Nguyen QT, Lee EJ, Huang MG, Park YI, Khullar A, Plodkowski RA (2015). Diagnosis and treatment of patients with thyroid cancer. Am Health Drug Benefits.

[6] Mohamed TY, Latif AM, Zaki AM, El Shahid MA, Ali MM, Lofty AE (2021). Accuracy of thyroid imaging reporting and data system in evaluation of thyroid neoplasm. Egyptian Journal of Hospital Medicine.

[7] Giordano D, Valcavi R, Thompson GB, Pedroni C, Renna L, Gradoni P, Barbieri V (2012). Complications of central neck dissection in patients with papillary thyroid carcinoma: results of a study on 1087 patients and review of the literature. Thyroid.

[8] Lee HS, Park C, Kim SW (2016). Primary tumour characteristics predict the invasiveness of lymph node metastases in papillary thyroid carcinoma patients. J Laryngol Otol.

[9] James BC, Mitchell JM, Jeon HD, Vasilottos N, Grogan RH, Aschebrook-Kilfoy B (2018). An update in international trends in incidence rates of thyroid cancer, 1973-2007. Cancer Causes Control.

[10] Levy I, Barki Y, Tovi F (1991). Giant cervical cyst: presenting symptom of an occult thyroid carcinoma. J Laryngol Otol.

[11] Vierhapper H, Niederle B, Bieglmayer C, Kaserer K, Baumgartner-Parzer S (2005). Early diagnosis and curative therapy of medullary thyroid carcinoma by routine measurement of serum calcitonin in patients with thyroid disorders. Thyroid.

[12] Sheils O (2005). Molecular classification and biomarker discovery in papillary thyroid carcinoma. Expert Rev Mol Diagn.

[13] Renshaw AA, Gould EW (2002). Why there is the tendency to "overdiagnose" the follicular variant of papillary thyroid carcinoma. Am J Clin Pathol.

[14] Harshan M, Crapanzano JP, Aslan DL, Vazquez MF, Saqi A (2009). Papillary thyroid carcinoma with atypical histiocytoid cells on fine-needle aspiration. Diagn Cytopathol.

[15] Trimboli P, Treglia G, Guidobaldi L (2015). Detection rate of FNA cytology in medullary thyroid carcinoma: a meta-analysis. Clin Endocrinol (Oxf).

[16] Haugen BR, Alexander EK, Bible KC (2016). 2015 American Thyroid Association management guidelines for adult patients with thyroid nodules and differentiated thyroid cancer: the American Thyroid Association guidelines task force on thyroid nodules and differentiated thyroid cancer. Thyroid.

[17] Yoon JH, Kim EK, Kwak JY, Moon HJ (2015). Effectiveness and limitations of core needle biopsy in the diagnosis of thyroid nodules: review of current literature. J Pathol Transl Med.

[18] Youssef Youssef, Abd-Elmonem Abd-Elmonem, M.H. M.H. The diagnostic value of ultrasonography in detection of different types of thyroid nodules. Egypt J Otolaryngol 36.

[19] Lee K, Anastasopoulou C, Chandran C, Cassaro S (2021). Thyroid cancer. StatPearls [Internet].

[20] Cawood TJ, Mackay GR, Hunt PJ, O'Shea D, Skehan S, Ma Y (2020). TIRADS management guidelines in the investigation of thyroid nodules; illustrating the concerns, costs, and performance. J Endocr Soc.

[21] Dy JG, Kasala R, Yao C, Ongoco R, Mojica DJ (2017). Thyroid imaging reporting and data system (TIRADS) in stratifying risk of thyroid malignancy at the Medical City. J ASEAN Fed Endocr Soc.

[22] Wettasinghe MC, Rosairo S, Ratnatunga N, Wickramasinghe ND (2019). Diagnostic accuracy of ultrasound characteristics in the identification of malignant thyroid nodules. BMC Res Notes.

[23] Ram N, Hafeez S, Qamar S, Hussain SZ, Asghar A, Anwar Z, Islam N (2015). Diagnostic validity of ultrasonography in thyroid nodules. J Pak Med Assoc.

[24] Yang GC, Fried K, Levine PH (2013). Detection of medullary thyroid microcarcinoma using ultrasound-guided fine needle aspiration cytology. Cytopathology.

[25] Bahaj AS, Alkaff HH, Melebari BN (2020). Role of fine-needle aspiration cytology in evaluating thyroid nodules. A retrospective study from a tertiary care center of Western region, Saudi Arabia. Saudi Med J.

